# A47 VIRTUAL REALITY SIMULATION TRAINING IN GASTROINTESTINAL ENDOSCOPY: A COCHRANE REVIEW

**DOI:** 10.1093/jcag/gwae059.047

**Published:** 2025-02-10

**Authors:** R Khan, N Sabrie, J Plahouras, B Johnston, M Scaffidi, S Grover, C Walsh

**Affiliations:** Division of Gastroenterology, Mayo Clinic Minnesota, Rochester, MN; University of Toronto, Toronto, ON, Canada; University of Toronto, Toronto, ON, Canada; Texas A&M University System, College Station, TX; Queen’s University, Kingston, ON, Canada; Scarborough Health Network, Scarborough, ON, Canada; The Hospital for Sick Children, Toronto, ON, Canada

## Abstract

**Background:**

Training with virtual reality (VR) simulation is increasingly used for health professions training to allow novices to practice in a learner-centered, risk-free environment. This review was performed to evaluate the effectiveness of VR simulation training in gastrointestinal endoscopy.

**Aims:**

To determine whether virtual reality simulation training can supplement and/or replace early conventional endoscopy training in diagnostic endoscopy for health professions trainees with limited or no prior endoscopic experience.

**Methods:**

We searched 17 databases from inception until October 18, 2023. We included randomised and quasi-randomised trials that compared VR simulation training with no training, conventional training, another form of simulation training, or an alternative method of VR training. We screened and abstracted data through Cochrane methodology. The primary outcome was composite score of competency. Secondary outcomes were procedure completion, procedure time, adverse events, patient discomfort, global rating of competency, and mucosal visualization. We calculated risk ratio for dichotomous outcomes and mean difference (MD) or standardised mean difference (SMD) for continuous outcomes with 95% CI. We used GRADE to assess the certainty of evidence.

**Results:**

We included 20 trials (500 participants; 3975 endoscopic procedures). There was insufficient evidence to determine the effect of VR training on composite score of competency compared to no training or conventional training. VR training was advantageous over no training based for independent procedure completion (RR 1.62, 95% CI 1.15 to 2.26; moderate certainty evidence), overall rating of performance (mean difference [MD] 0.45, 95 %CI 0.15 - 0.75, very low certainty evidence), and mucosal visualization (MD 0.60, 95 %CI 0.20 - 1.00, very low certainty evidence). VR training resulted in fewer independent procedure completions compared to conventional training (RR = 0.45, 95 %CI 0.27 - 0.74, low certainty evidence). We found no differences between VR training and no training or conventional training for other outcomes. Based on qualitative analysis, we found no significant differences between VR training and other forms of simulation training. VR curricula based in educational theory provided benefit with respect to composite score of competency, compared with unstructured curricula. Based on qualitative analysis, we found no significant differences between VR training and other forms of simulation training. VR curricula based in educational theory provided benefit with respect to composite score of competency, compared with unstructured curricula.

**Conclusions:**

VR simulation training in endoscopy can supplement conventional endoscopy training and provides benefit compared to no training.

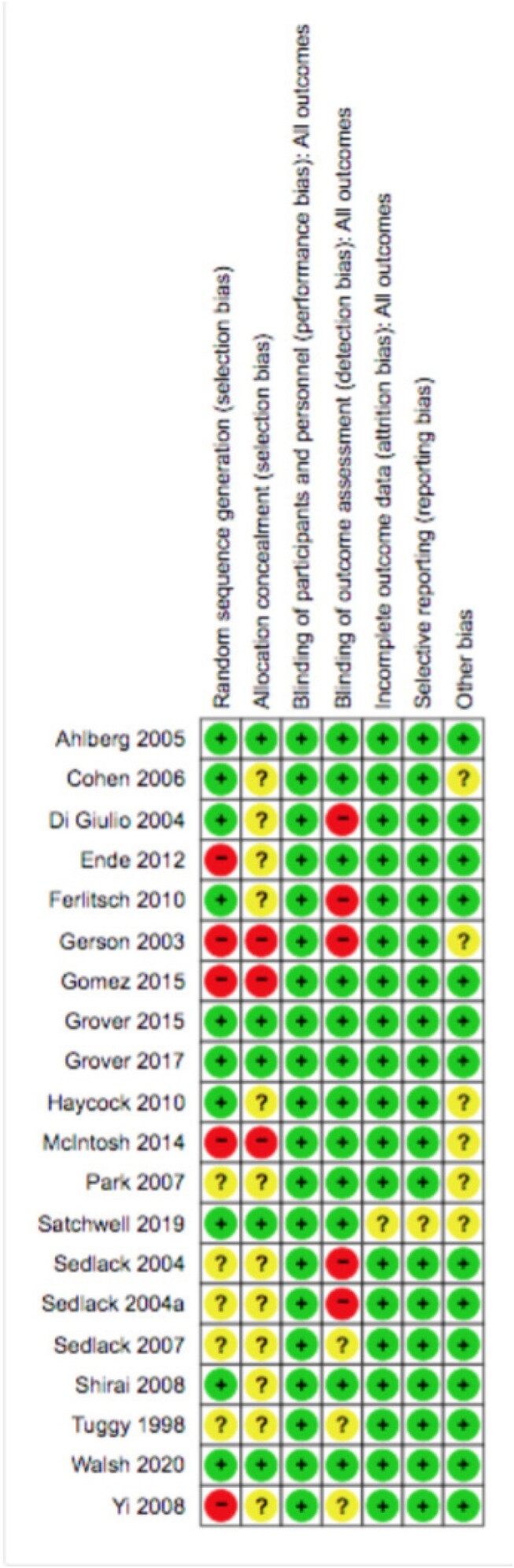

Risk of bias summary

**Funding Agencies:**

None

